# Femtosecond thin-flap laser assisted in situ keratomileusis for correction of post-penetrating keratoplasty ametropia: long-term outcome

**DOI:** 10.1186/s12886-024-03428-3

**Published:** 2024-04-16

**Authors:** Bahram Einollahi, Javad Rezaei, Mohammad-Mehdi Sadoughi, Sepehr Feizi, Neda Einollahi, Amir Reza Veisi, Kiana Hassanpour

**Affiliations:** 1 Ophthalmic Research Center, Research Institute for Ophthalmology and Vision Science, Shahid Beheshti University of Medical Sciences, Tehran, Iran; 2 Department of Ophthalmology, Labbafinejad Medical Center, Shahid Beheshti University of Medical Sciences, 23rd PaidarFard St., Boostan 9, Pasdaran Ave, Tehran, 16666 Iran

**Keywords:** Keratoconus, Penetrating keratoplasty, Refractive error, Femtosecond thin-flap LASIK, Long-term outcomes

## Abstract

**Purpose:**

To evaluate the long-term clinical outcomes of femtosecond thin-flap LASIK (femto-LASIK) for correction of refractive error after penetrating keratoplasty in keratoconus-affected eyes.

**Setting:**

a private ophthalmology clinic.

**Design:**

Prospective interventional case series.

**Methods:**

This prospective interventional case series enrolled 22 eyes of 22 patients who underwent femto-LASIK for the management of post-penetrating keratoplasty ametropia. The refractive error, uncorrected (UDVA), and corrected (CDVA) distance visual acuities and vector analysis were reported in short-term and long-term period after surgery.

**Results:**

The mean age was 32.7 ± 7.5 years (range, 23 to 47 years) at the surgery time. The average time between PK and femto-LASIK was 42.5 ± 31.7 months. The average follow-up duration after femto-LASIK was 81.2 ± 18.6 months. The mean preoperative UDVA significantly improved from 0.47 ± 0.15 logMAR to 0.35 ± 0.14 logMAR at 12 months (*P* = 0.048) and 0.4 ± 0.17 at final follow-up exam (*P* = 0.007). CDVA was 0.22 ± 0.1 at baseline which improved to 0.18 ± 0.15 and 0.15 ± 0.1 logMAR at 12 and 81 months, respectively. (Ps = 0.027, 0.014). The mean cylinder before surgery was − 5.04 ± 1.4D which significantly decreased to -1.5 ± 0.8 D at 12 months postoperatively. (*P* < 0.001). There was a significant increase in refractive astigmatism from 12 months to 81 months postoperatively (-3.1 ± 2.0, *P* = 0.002). At the final visit, the efficacy index was 0.83, and the safety index was 1.16.

**Conclusions:**

Despite the short-term outcome indicated that femo-LASIK was effective for correction of post-keratoplasty ametropia during short-term period, a notable regression in its effect was observed in the long-term follow-up. Therefore, the predictability of this technique might decrease in the long-term.

**Supplementary Information:**

The online version contains supplementary material available at 10.1186/s12886-024-03428-3.

## Introduction

Penetrating keratoplasty (PK) remains a frequent surgical method to improve the visual acuity of patients with corneal opacity, keratoconus, or other corneal pathologies. Since the chance of postoperative corneal transparency had significantly increased with the use of pharmacological therapies, refractive errors are the most common cause of reduced visual acuity and patient dissatisfaction [1, 2]. It is estimated that 15–31% of patients who undergo PK develop an astigmatism of > 5 diopters [3]. In addition to astigmatism, incidence of spherical refractive errors are also common after PK [3]. 

Various nonsurgical measures including spectacles, and soft and hard contact lenses can correct post-PK ametropia. However, incisional or laser vision correction (LVC) methods are available surgical methods when noninvasive measures fail to correct post-PK refractive error [4]. Among LVCs, both photorefractive keratectomy (PRK) and laser in situ keratomileusis (LASIK) can be used [5]. Despite the improvement of PRK outcome with the use of mitomycin-C (MMC) and customized wave-front or topography-guided ablations, there are still concerns about the risk of corneal haziness and unpredictable refractive results [6, 7].

Several studies demonstrated the advantages of LASIK to correct refractive errors following PK [8]. However, some complications are also reported including a lamellar cut at the donor-recipient junction point, wound dehiscence, alterations in the astigmatism axis, and epithelial ingrowth, under correction and long-term intrinsic graft instability [9–11]. 

Several studies suggest that using a femtosecond laser for flap creation during the LASIK procedure (femto-LASIK) offers several advantages over microkeratome including lower rates of button-hole and free cap formation and more precise flap thickness and diameter. In addition, better visual outcomes, lower rates of higher-order aberrations, and less severe dry eye have been reported in femto-LASIK compared to conventional LASIK [12, 5]. While both microkeratome and Femto-LASIK have been shown to be effective in correcting post-PK refractive errors, Femto-LASIK has several advantages, including more predictable flap thickness and planarity, the ability to choose flap centration, and fewer intraoperative complications [13]. 

There are studies reporting the short-term results of femto-LASIK for the correction of refractive errors after corneal transplantation [12, 5]. To the best of our knowledge, studies reporting the outcome of LASIK after PK are short and middle-term. However, the long-term outcome is of utmost importance. It is possible that the corneal remodeling specially at the junction of the graft will reduce the efficacy of the procedure. Long-term follow-up allows for the assessment of the stability and predictability of the refractive outcomes, ensuring that the desired visual outcomes are maintained over time. In the present study, we report the long-term outcomes of femtosecond thin-flap LASIK for correction of refractive error after PK.

## Materials and methods

This prospective interventional case series enrolled consecutive keratoconus-affected patients who underwent femto-LASIK for the treatment of post-PK ametropia between 2011 and 2018. The study protocol was approved by the Institutional Review Board affiliated with the Shahid Beheshti University of Medical Sciences in Tehran, Iran. The protocol of the study adhered to the declaration of Helsinki, and an informed consent was obtained from all patients.

### Inclusion and exclusion criteria

The inclusion criteria included a history of PK performed for the management of keratoconus and complete suture removal at least 18 and 12 months before enrollment, respectively. Other inclusion criteria were stable refraction and corneal topography indices for the previous 6 months, astigmatism < 6 diopters, central corneal pachymetry > 500 microns, and presence of regular astigmatism based on corneal topography. The residual corneal thickness was planned to be more than 300 microns in all patients.

Exclusion criteria included wound healing disorders, moderate to severe dry eye, collagen vascular diseases, history of herpes simplex keratitis, graft edema, insufficient healing of the graft-host junction, any signs of inflammation or vascularization or corneal ectasia in corneal graft and paraclinical, instability of refraction, any signs of rejection or decompensation of the corneal graft, any surgery performed after PK (astigmatic keratotomy (AK) or any kind of relaxing incisions, excimer laser surgery, resuturing, intrastromal corneal ring segment (ICRS)).

### Surgical technique

A single experienced surgeon (B.E.) performed all surgeries. All patients underwent femto-Lasik under topical anesthesia. Flaps were created with an LDV Z6 (Ziemer Ophthalmic Systems AG, Port, Switzerland). The created flaps had diameters of 8.5 to 9 mm, thickness of 100–110 microns, standard 12-o’clock position, and 45° side-cut angles. The hinges were set in a superior orientation with a hinge length of 4.0 mm. The flap was separated and slowly lifted away centrifugally. Stromal tissue ablation was performed with the excimer laser using a tissue-saving function (Technolas 217-Z, Excimer laser, Baush & Lomb) laser as a wave-front optimized laser ablation. Subjective refraction was used in all cases, and the target refraction was set at emmetropia. The ablation zone was 8.5–9 mm. Postoperative topical medication regimens included betamethasone eye drops 4 times per day for 7 days which were tapered over 6 to 8 weeks, and chloramphenicol eye drops 4 times per day for 7 days.

### Ophthalmic examination

Baseline demographic and clinical data were collected for all study participants. At baseline, all patients underwent a comprehensive ophthalmic examination including uncorrected (UDVA) and corrected (CDVA) distance visual acuities, manifest and cycloplegic refraction, spherical equivalent (SE), slit-lamp examination, Goldmann applanation tonometry, and dilated fundus examination.CDVA was measured using Snellen chart from standard 6 m distance. Corneal topography (Tomey TMS4: Topographic Modelling System, Tomey Corporation) was used to measure corneal astigmatism, and corneal pachymetry (Tomey pachymeter SP3000, Tomey Corporation) was used to measure central graft thickness.

Postoperatively, patients were visited on days 1, 3, and 7 after surgery regarding complications like infection, and flap related complications, then monthly for up to 6 months, and then every 6 months in terms of refractive error, keratometry and complications. Each postoperative visit included UDVA, CDVA, slit lamp examination, IOP, funduscopy and corneal topography.

### Statistical analysis

The primary outcome measure was UDVA and CDVA. Safety and efficacy indices were calculated. Safety Index was defined as CDVA after treatment divided by CDVA before treatment The Efficacy Index was defined as UCVA after treatment divided by CDVA before treatment. To describe data, we used mean, SD, median and range, frequency, and percentage values. Paired t-test was used to compare the results. Vector analysis was performed using the Alpins method [14]. All data were analyzed using SPSS software (IBM Corp. Released 2019. IBM SPSS Statistics for Windows, Version 24.0. Armonk, NY: IBM Corp) The significance level was considered to be *p* < 0.05.

## Results

Twenty-two eyes of 22 consecutive patients (11 males and 11 females) who met the inclusion criteria underwent femto-LASIK. The mean patient age was 32.7 ± 7.5 years (range, 23 to 47 years) at the surgery time. Eighteen patients completed the last follow-up (81 months) but 4 patients were lost to follow-up after 12 months after femto-LASIK and data on these patients is not available. The clinical outcome is reported in two follow-up periods of 12 months (for 22 eyes) and 81 months (for 18 eyes).

The average time interval between PK and femto-LASIK was 42.5 ± 31.7 months (range, 20 to 144 months). Preoperative UDVA was 0.48 ± 0.15 logMAR. The average keratometry before femto-LASIK was 45.2 ± 1.7 (range, 42.1–49.2). The average follow-up duration after femto-LASIK was 81.2 ± 18.6 ranging from 48 to 132 months. None of the patients had preoperative cataract, retinal or optic nerve problems (Table 1).

**Table 1:** Preoperative information of eyes

	Number of eyes = 22	
Variable	Mean ± SD	Median
Age (years)	32.7 ± 7.5	31
PK to femto-LASIK (months)	42.5 ± 31.7	33.5
FU (months)	81.2 ± 18.6	79
Preop Mean K	45.2 ± 1.7	45.13
Preop Astigmatism	5.1 ± 1.4	− 5.75
Preop Spherical equivalent	-3.1 ± 2.8	− 3.5

PK: Penetrating keratoplasty; K: keratometry Preop: Preoperative; FU: Follow-up

The average preoperative CDVA was 0.22 ± 0.1 logMAR (range, 0–0.39). The average preoperative SE was − 3.03 ± 2.8 D (range, -8.0 to + 3.0). The mean refractive astigmatism was 5.04 ± 1.4 D (range, − 6.0 to -1.50). (Table 1)

82% of eyes (18/22) had preoperative cylinders of ≥ 4.00 D. No intraoperative or postoperative complications were encountered during the study period.

### Refractive outcome

Preoperative UDVA significantly improved from 0.47 ± 0.15 logMAR preoperatively to 0.35 ± 0.14 logMAR at 12 months (*P* = 0.048) and 0.4 ± 0.17 logMAR at 81 months (*P* = 0.007) postoperatively. CDVA increased from 0.22 ± 0.1 logMAR preoperatively to 0.18 ± 0.15 logMAR (*P* = 0.027) and 0.15 ± 0.1 logMAR (*P* = 0.014) at 12 and 81 months postoperatively, respectively.

**Fig. 1:**
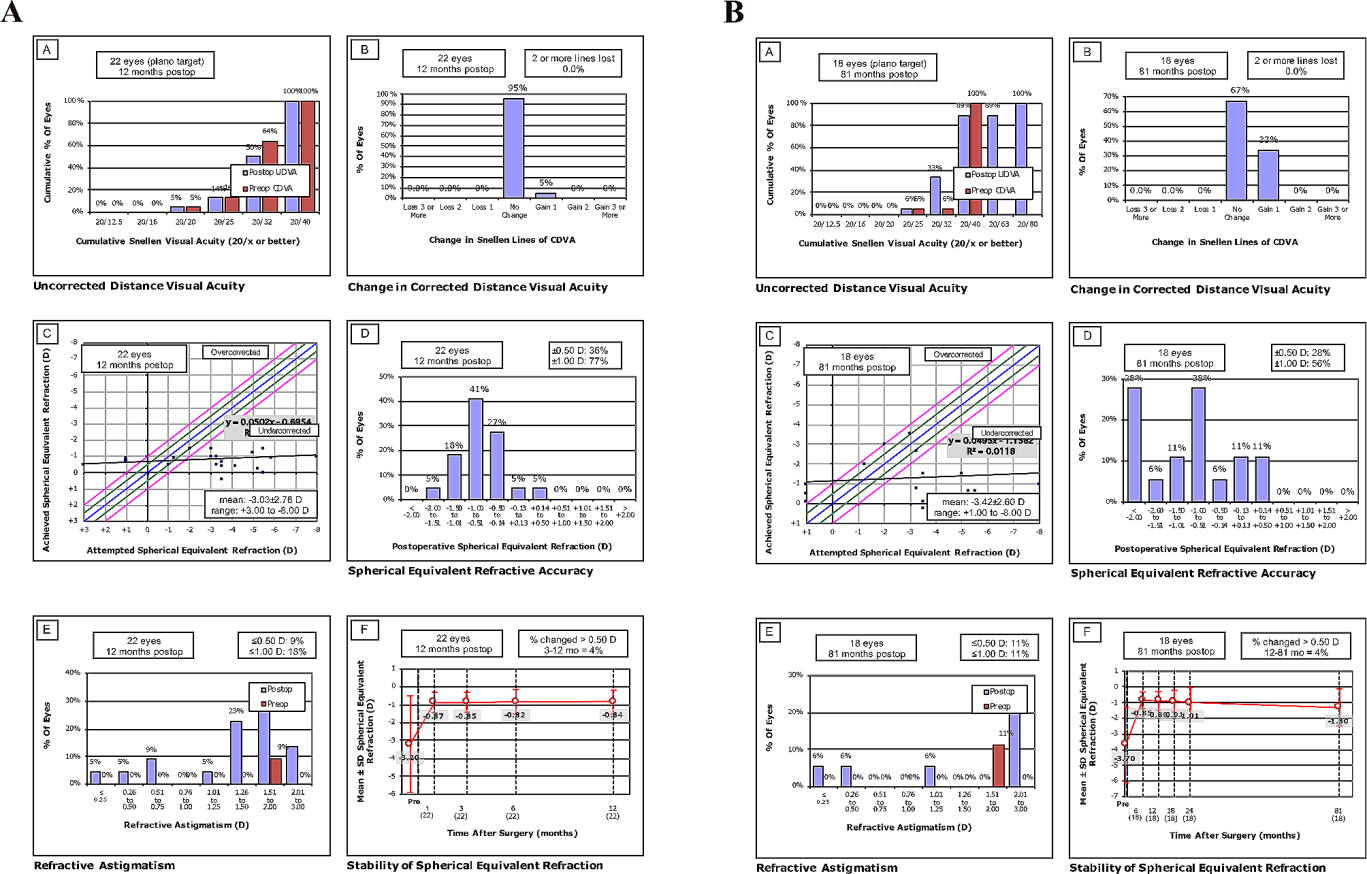
The standard 6 graphs reporting the refractive outcomes in the 12th months (**A**). The standard 6 graphs reporting the refractive outcomes in the 81st months (**B**)

The average SE was − 3.03 ± 2.8D preoperatively which significantly decreased to -0.84 ± 0.67 D at postoperative month 12 (*P* < 0.001) and − 1.3 ± 1.2 D at postoperative month 81 (*P* < 0.001). A borderline difference was observed between SE at postoperative months 12 and 81 (*P* = 0.055). Tables 2 and 3 present the results for myopic and hyperopic treatment separately. At 12 months, SE was within ± 0.50 D of emmetropia in 36% of eyes and within ± 1.00 D of emmetropia in 77% of eyes. These figures were 28% and 56% at postoperative month 81, respectively. (Fig. 1)

**Table 2:** Refractive outcome of the eyes undergoing myopic correction

Variable	Pre (*N* = 15)		Month 12 (*N* = 15)		P	Month 81 (*N* = 13)		P From Baseline
	Mean ± SD	Median	Mean ± SD	Median		Mean ± SD	Median	
Sphere	-2.1 ± 1.8	-2	-0.08 ± 0.6	0.0	< 0.001	0.04 ± 1.25	0.0	0.002†
Cylinder	-4.9 ± 1.5	-5.50	-1.50 ± 0.8	-1.50	< 0.001	-2.73 ± 1.8	-3.00	0.001†
SE	-4.5 ± 1.5	-4.12	-0.86 ± 0.8	-0.87	< 0.001	-1.33 ± 1.2	-1.00	< 0.001†
CDVA	0.21 ± 0.11	0.22	0.18 ± 0.16	0.15	0.043	0.15 ± 0.11	0.15	0.037‡
UDVA	0.47 ± 0.16	0.39	0.37 ± 0.16	0.3	0.03	0.4 ± 0.17	0.3	0.04‡

CDVA: corrected distance visual acuity; UDVA: Uncorrected distance visual acuity † Based on paired t-test. ‡ Based on Wilcoxon singed-rank test. SD: Standard deviation; SE: Spherical equivalent

**Table 3:** Refractive outcome of eyes undergoing hyperopic correction

Variable	Pre (*N* = 7)		Month 12 (*N* = 7)		P	Month 81 (*N* = 5)		P From baseline
	Mean ± SD	Median	Mean ± SD	Median		Mean ± SD	Median	
Sphere	2.89 ± 1.8	2.75	− 0.07 ± 0.2	0	0.001†	0.75 ± 1.4	0.25	0.001†
Cylinder	-5.30 ± 0.9	-6.00	-1.50 ± 0.9	-1.5	0.001†	-4.15 ± 2.56	-4.00	0.32†
SE	0.25 ± 1.7	0.50	-0.82 ± 0.3	− 0.75	0.04†	-1.30 ± 1.2	-1.00	0.04†
CDVA	0.24 ± 0.11	0.22	0.18 ± 0.16	0.15	0.03‡	0.18 ± 0.06	0.22	0.03‡
UDVA	0.49 ± 0.14	0.52	0.32 ± 0.11	0.3	0.04 ‡	0.34 ± 0.08	0.39	0.45‡

CDVA: corrected distance visual acuity; UDVA: Uncorrected distance visual acuity. † Based on paired t-test. ‡ Based on Wilcoxon singed-rank test. SD: Standard deviation; SE: Spherical equivalent

The mean preoperative refractive astigmatism decreased from − 5.04 ± 1.4 D to -1.5 ± 0.8 D at postoperative month 12 (*P* < 0.001) and − 3.1 ± 2.0 D at postoperative month 81 (*P* = 0.035). Compared to the value measured at postoperative month 12, there was a significant increase in refractive astigmatism at the final follow-up exam (*P* = 0.002). (Tables 2 and 3, Supplementary Tables 1, 2)

Figure 1 shows the graphs for standard reporting outcomes of refractive surgery after 12 months (A) and 81 months (B).

### Safety, efficacy, and complications

UDVA showed improvement in all participants. Similarly, post-operative CDVA in all eyeswas equal to or more than the pre-operative CDVA, and none of the eyes lost any line of the Snellen chart. At the final visit, the efficacy index was 0.83, and the safety index was 1.16. We observed no specific complications related to femto-LASIK or graft including rejection, vascularization, infection, or epithelial ingrowth. Seven patients required spectacles or contact lenses to enhance their vision, and one patient had to undergo relaxing incisions and compression sutures due to high astigmatism.

### Vector analysis

**Fig. 2:**
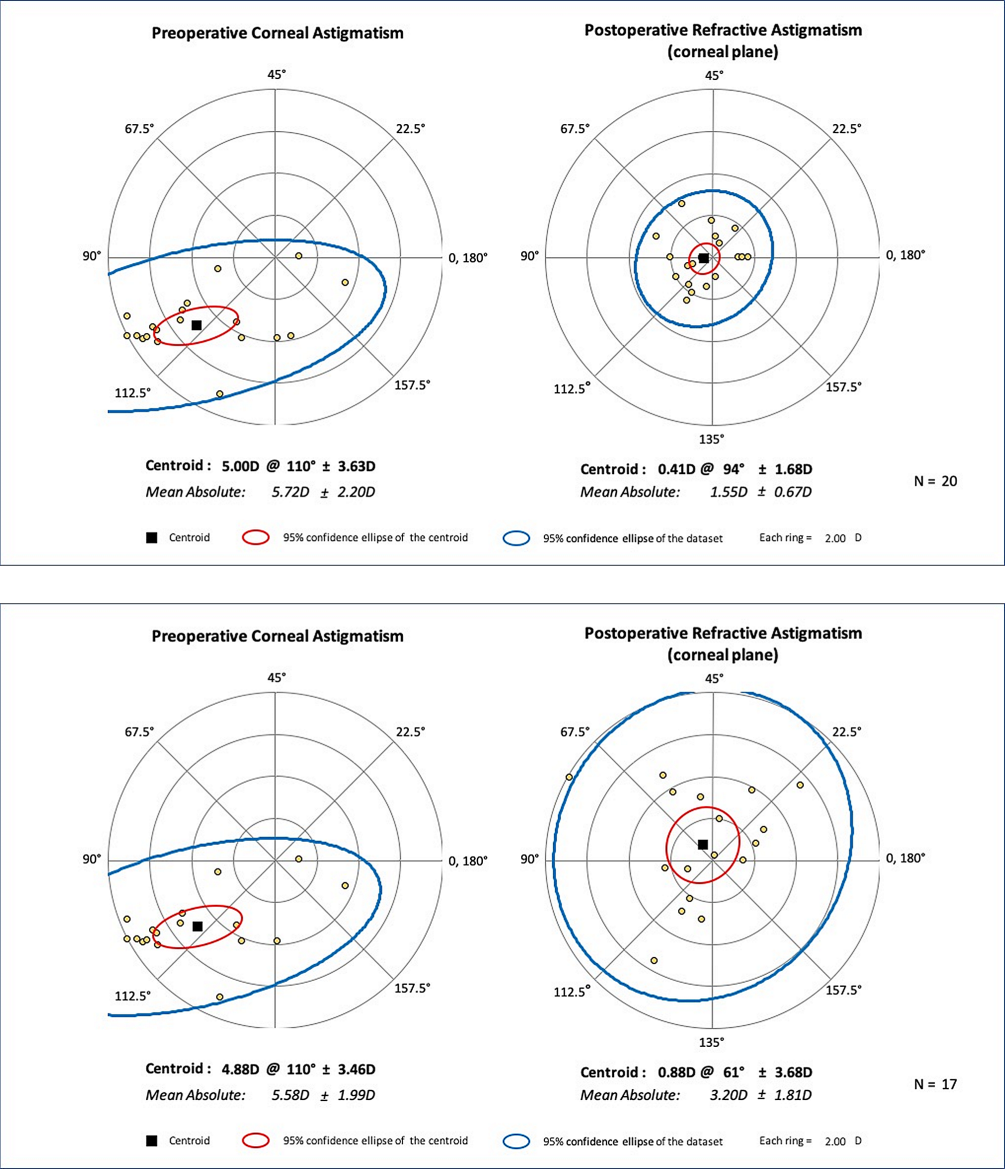
The double plot showing preoperative corneal astigmatism and postoperative refractive astigmatism at the corneal plane at month 12 (**A**) and month 81 **(B**). Each ring demonstrates 2 diopters of astigmatism

At 12 months follow-up, mean target-induced astigmatism (TIA) and surgically-induced astigmatism (SIA) was 4.7 ± 1.8, and 4.1 ± 2.0. The mean angle of error was 1.29 ± 11.8 degrees. The corresponding values at month 81 was 3.2 ± 1.2 and 3.6 ± 1.7, respectively. Figures 2 and 3 present double-angle plots calculated at the cornea plane at two-time follow-up intervals. (Fig. 2)

## Discussion

The present study evaluated long-term safety and efficiency of femtosecond thin-flap LASIK for correcting post-PK ametropia in keratoconus. Compared to previous studies that reported short-term results, the present study reports outcomes measured 48 to 132 months postoperatively.

Mild to moderate post-PK refractive error can often be corrected with spectacles or contact lenses. In cases with anisometropia or high refractive error, where contact lens wear is not an option, corrective refractive surgery can be considered as a viable means to achieve emmetropia [15]. 

There are several methods available for addressing post-keratoplasty ametropia, including photorefractive keratectomy (PRK), trans-PRK, conventional LASIK, and femto-LASIK. In a recent meta-analysis comprising 31 studies and involving 732 eyes, it was determined that approximately 50% of cases achieved the desired refractive outcome within a range of 1.0 diopter (D) across all treatment modalities. The overall incidence of a decline in corrected distance visual acuity (CDVA) by 2 lines or more was found to be 5.8%. Notably, the main adverse effects that could affect visual acuity were corneal haze and regression for PRK, and epithelial ingrowth for LASIK. This meta-analysis demonstrated that the outcomes of PRK and LASIK were comparable, although there was a slightly higher incidence of corneal haze following PRK, indicating a relatively worse outcome in this regard [13]. 

The correction of ametropia after PK with LASIK was first reported by Arenas and Maglione in 1997 [16]. Compared to PRK, LASIK offers faster visual recovery, less scarring, less regression, and can correct a greater range of refractive errors [3]. Alio JL et al. reported on the visual and refractive outcomes of LASIK performed in either one or two steps (lamellar cut followed by ablation in one or two procedures) after a six-month follow-up. They concluded that the two-step technique improved the accuracy of excimer laser correction of post-PK astigmatism [17]. However, LASIK has limitations, including its ability to correct astigmatism and its potential for complications such as epithelial ingrowth, buttonhole, free or incomplete flaps, an increased risk of photoablation-induced graft rejection, and diminished flap adhesion [3]. 

In 2010, Barequet et al. first reported on the use of femtosecond thin-flap LASIK to correct ametropia after PK [5]. Their study included 11 eyes and had a follow-up of six months. In a study by Ghoreishi et al., 34 eyes underwent Femto-LASIK for refractive error correction after PK, with a follow-up period of 12 months. The results were considered safe, effective, and predictable, and were deemed acceptable during the 12-month follow-up [12]. 

Our study, like previous studies, [5, 12, 15, 18–19] shows the safety and efficacy of femto-LASIK in correcting refractive error after PK. (Supplementary Table 3) Intraoperatively, there were no complications observed during flap creation and laser ablation. Additionally, no wound-healing issues, graft rejection, or ectasia were reported during the follow-up period. The results demonstrated a significant improvement in UDVA, CDVA, SE, and cylinder.

Astigmatism control after PK is complicated and should be decided on a case by case basis. Selecting a larger graft size (more than 8 mm) during keratoplasty might enhance the predictability due to removing thin cornea and placing the host junction outside the flap.

Eighteen patients attended the final follow-up, whereas four were lost to follow-up after 12 months of Femto-LASIK, and their data was not available. In the long run, seven patients required spectacles or contact lenses to enhance their vision, and one patient had to undergo relaxing incisions and compression sutures due to high astigmatism. Nevertheless, despite these issues, the long-term results demonstrated an improvement in UDVA, CDVA, SE, and astigmatism compared to the preoperative values, although the efficacy of the surgery was lower than that in the short-term follow-up.

The study has a limitation that should be considered when interpreting the results, namely the small sample size and the fact that 4 patients were lost to follow-up. However, the strength of the present study includes report of the long-term refractive outcomes after femto-LASIK in patients with ametropia after PK.

In summary, Despite the short-term outcome indicated that femo-LASIK was effective for correction of post-keratoplasty ametropia during short-term period, a notable regression in its effect was observed in the long-term follow-up. Therefore, further long-term studies, including a larger number of patients, are necessary to evaluate the efficacy of this surgical procedure.

### Electronic supplementary material

Below is the link to the electronic supplementary material.

Supplementary Material 1

## Data Availability

The data is available upon the request from the corresponding author.
