# Supplementary material for: Femtosecond thin-flap laser assisted in situ keratomileusis for correction of post-penetrating keratoplasty ametropia: long-term outcome
Source: BMC Ophthalmol. 2024 Apr 16;24:174. doi: 10.1186/s12886-024-03428-3 (PMC11020473; doi:10.1186/s12886-024-03428-3)
Supplement: Supplementary file 1 — Supplementary Material 1 [file 12886_2024_3428_MOESM1_ESM.docx]

**Supplementary Table 1.** **Preoperative and postoperative refraction, UDVA and CDVA in patients undergoing myopic correction.**

|  |  | **Before LASIK** | | |  | **Month 12** | | |  | **Month 81** | | |
| --- | --- | --- | --- | --- | --- | --- | --- | --- | --- | --- | --- | --- |
| **Patient** | **Age** | **Refraction** | **UDVA** | **CDVA** |  | **Refraction** | **UDVA** | **CDVA** |  | **Refraction** | **UDVA** | **CDVA** |
| #1 | 31 | plano -6 * 105 | 20/100 | 20/50 |  | -0.75 -1.5 * 70 | 20/50 | 20/32 |  | NA | NA | NA |
| #2 | 47 | Plano -6 * 10 | 20/70 | 20/32 |  | Plano -2 * 30 | 20/40 | 20/32 |  | -0.5 -6 * 30 | 20/50 | 20/32 |
| #3 | 32 | -1.5 -5.25 * 135 | 20/70 | 20/32 |  | + 0.5 -1.75 * 135 | 20/32 | 20/25 |  | NA | NA | NA |
| #4 | 31 | -0.5 -6 * 120 | 20/50 | 20/32 |  | +1 -1.25 * 90 | 20/32 | 20/32 |  | +3 - 5.5 * 110 | 20/80 | 20/40 |
| #5 | 30 | -3.75 -3 * 5 | 20/40 | 20/25 |  | plano -0.5 * 90 | 20/25 | 20/20 |  | -0.5 -0.25 * 120 | 20/32 | 20/25 |
| #6 | 29 | -3.25 -5.25 * 20 | 20/50 | 20/32 |  | plano -1.75 * 25 | 20/32 | 20/25 |  | -1 -3 * 30 | 20/32 | 20/25 |
| #7 | 40 | -0.75 -5.5 * 15 | 20/70 | 20/32 |  | plano -1.25 * 10 | 20/40 | 20/32 |  | +1 -2.25 * 5 | 20/50 | 20/32 |
| #8 | 25 | -2.5 -6 * 140 | 20/70 | 20/32 |  | + 1 - 2 * 180 | 20/40 | 20/32 |  | +1.75 -3 * 140 | 20/50 | 20/32 |
| #9 | 24 | -1 -4.5 * 45 | 20/70 | 20/32 |  | plano -2 * 15 | 20/32 | 20/25 |  | -1.5 -2.25 * 20 | 20/32 | 20/25 |
| #10 | 27 | -2.75 -1.5 * 80 | 20/32 | 20/20 |  | plano -0.75 * 120 | 20/20 | 20/20 |  | Plano -3 * 105 | 20/32 | 20/20 |
| #11 | 29 | -0.25 -6 * 155 | 20/70 | 20/40 |  | plano -1 * 120 | 20/40 | 20/32 |  | +0.25 -2 * 130 | 20/40 | 20/32 |
| #12 | 26 | -3 -5 * 20 | 20/50 | 20/32 |  | -1 -1 * 10 | 20/32 | 20/32 |  | plano -1.25 *10 | 20/40 | 20/32 |
| #13 | 37 | -2 -6 * 35 | 20/70 | 20/40 |  | plano -2.5 * 30 | 20/32 | 20/25 |  | plano -3 * 40 | 20/32 | 20/20 |
| #14 | 47 | -3.25 -6 170 | 20/100 | 20/50 |  | -1 -3 * 170 | 20/63 | 20/50 |  | -1 -4 * 150 | 20/100 | 20/50 |
| #15 | 31 | -7 -2 * 30 | 20/70 | 20/40 |  | -1 | 20/40 | 20/32 |  | -1 | 20/40 | 20/32 |

**Supplementary Table 2.** Preoperative and postoperative refraction, UDVA and CDVA in patients undergoing hyperopic correction.

|  |  | **Before LASIK** | | |  | **Month 12** | | |  | **Month 81** | | |
| --- | --- | --- | --- | --- | --- | --- | --- | --- | --- | --- | --- | --- |
|  | **Age** | **Refraction** | **UDVA** | **CDVA** |  | **Refraction** | **UDVA** | **CDVA** |  | **Refraction** | **UDVA** | **CDVA** |
|  |  |  |  |  |  |  |  |  |  |  |  |  |
| #1 | 40 | +4 - 6 * 160 | 20/100 | 20/50 |  | plano -1.5 * 40 | 20/50 | 20/32 |  | +3 -8 * 165 | 20/50 | 20/32 |
| #2 | 23 | +1.75 -6 * 155 | 20/70 | 20/32 |  | plano -1.75 * 135 | 20/32 | 20/32 |  | plano -4 * 120 | 20/50 | 20/32 |
| #3 | 40 | 3.5 -5 * 170 | 20/70 | 20/32 |  | plano -1.75* 90 | 20/40 | 20/32 |  | +1 -2.25 * 100 | 20/50 | 20/32 |
| #4 | 45 | +6 -6 * 150 | 20/70 | 20/40 |  | -0.5 | 20/40 | 20/32 |  | NA | NA | NA |
| #5 | 34 | 1.25 -4.5 * 50 | 20/40 | 20/25 |  | plano -1 * 50 | 20/25 | 20/20 |  | NA | NA | NA |
| #6 | 27 | 1 -6 * 150 | 20/70 | 20/32 |  | plano -3 * 150 | 20/32 | 20/25 |  | -0.5 -5 * 150 | 20/50 | 20/25 |
| #7 | 26 | 2.75 -3.5 * 80 | 20/70 | 20/40 |  | plano -1.5 * 90 | 20/40 | 20/32 |  | +0.25 -1.5 * 90 | 20/40 | 20/32 |

**Supplementary Table 3.** Summary of studies reporting the clinical outcome of Femto-LASIK after corneal transplantation.

| Study | Year | No. of eyes | Follow-up Duration  (Months) | Type of surgery | Preoperative  Cylinder (Average ± SD) | Postoperative Cylinder(Average ± SD) |
| --- | --- | --- | --- | --- | --- | --- |
| Barequet et al (6) | 2010 | 11 eyes | 6 m | PKP | -6.60±3.60 | -2.90±2.00 |
| Ghoreishi et al (5) | 2013 | 34 eyes | 12 m | PKP | -4.60±2.40 | -2.10±1.30 |
| Balestrazzi et al (13) | 2017 | 13 eyes | 6 m | DALK | -4.35±1.90 | -0.92±1.39 |
| Belen Alfonsa-Bartolozzi et al (14) | 2022 | 10 eyes | 36-60 m | DALK | -3.88±1.00 | 0.93±0.39 |
